# Supplementary material for: Multidimensional memory topography in the medial parietal cortex identified from neuroimaging of thousands of daily memory videos
Source: Nat Commun. 2022 Oct 31;13:6508. doi: 10.1038/s41467-022-34075-1 (PMC9622880; doi:10.1038/s41467-022-34075-1)
Supplement: Supplementary file 2 — Reporting Summary [file 41467_2022_34075_MOESM2_ESM.pdf]

nature portfolio

Corresponding author(s): Wilma A. Bainbridge

Last updated by author(s): Sep 28, 2022

Reporting Summary

Nature Portfolio wishes to improve the reproducibility of the work that we publish. This form provides structure for consistency and transparency in reporting. For further information on Nature Portfolio policies, see our Editorial Policies and the Editorial Policy Checklist.

Statistics

For all statistical analyses, confirm that the following items are present in the figure legend, table legend, main text, or Methods section.

n/a	Confirmed	
☐	☒	The exact sample size (n) for each experimental group/condition, given as a discrete number and unit of measurement
☐	☒	A statement on whether measurements were taken from distinct samples or whether the same sample was measured repeatedly
☐	☒	The statistical test(s) used AND whether they are one- or two-sided<br>*Only common tests should be described solely by name; describe more complex techniques in the Methods section.*
☐	☒	A description of all covariates tested
☐	☒	A description of any assumptions or corrections, such as tests of normality and adjustment for multiple comparisons
☐	☒	A full description of the statistical parameters including central tendency (e.g. means) or other basic estimates (e.g. regression coefficient) AND variation (e.g. standard deviation) or associated estimates of uncertainty (e.g. confidence intervals)
☐	☒	For null hypothesis testing, the test statistic (e.g. F, t, r) with confidence intervals, effect sizes, degrees of freedom and P value noted<br>*Give P values as exact values whenever suitable.*
☒	☐	For Bayesian analysis, information on the choice of priors and Markov chain Monte Carlo settings
☐	☒	For hierarchical and complex designs, identification of the appropriate level for tests and full reporting of outcomes
☐	☒	Estimates of effect sizes (e.g. Cohen's d, Pearson's r), indicating how they were calculated

Our web collection on statistics for biologists contains articles on many of the points above.

Software and code

Policy information about availability of computer code

Data collection	Data was collected using Psychtoolbox-3 for MATLAB and the cellphone app 1 Second Everyday.
Data analysis	Data was analyzed using MATLAB R2019a, and AFNI and SUMA versions 22.2.12.

For manuscripts utilizing custom algorithms or software that are central to the research but not yet described in published literature, software must be made available to editors and reviewers. We strongly encourage code deposition in a community repository (e.g. GitHub). See the Nature Portfolio guidelines for submitting code & software for further information.

Data

Policy information about availability of data

All manuscripts must include a data availability statement. This statement should provide the following information, where applicable:

- Accession codes, unique identifiers, or web links for publicly available datasets
- A description of any restrictions on data availability
- For clinical datasets or third party data, please ensure that the statement adheres to our policy

Video features, memory features, and fMRI data generated in this study have been deposited in a repository on the Open Science Framework under access link https://osf.io/exb7m/ . Raw memory videos and memory geocoordinate information are protected and are not available due to data privacy laws. The graph data generated in this study are provided in the Source Data file.

Human research participants

Policy information about studies involving human research participants and Sex and Gender in Research.

Reporting on sex and gender	14 female and 9 male human adults participated in the study. Sex was self-reported by participants. Analyses were not separated by sex, due to no hypothesized differences by sex.
Population characteristics	Healthy adults (Mean age=28.3, SD=5.86, Range=19 to 37.1 years) were recruited for the study. All participants were right-handed, with normal or corrected vision, and had at least 6 months of recorded memory videos to participate in the study.
Recruitment	In order to recruit participants for this study, the 1SE app posted an in-app advertisement for users with IP addresses in the Washington, DC area to participate in a study based around their memories. The individuals who use this app may not be representative of the general US adult population, and there is likely a self-selection bias of participants who are motivated to use the app for 6+ months and take the time to participate in an MRI study.
Ethics oversight	Participants consented to participating in the study and having their video data used in the study, following the guidelines of the NIH Institutional Review Board (NCT00001360, 93M-0170).

Note that full information on the approval of the study protocol must also be provided in the manuscript.

Field-specific reporting

Please select the one below that is the best fit for your research. If you are not sure, read the appropriate sections before making your selection.

☐ Life sciences ☒ Behavioural & social sciences ☐ Ecological, evolutionary & environmental sciences

For a reference copy of the document with all sections, see nature.com/documents/nr-reporting-summary-flat.pdf

Behavioural & social sciences study design

All studies must disclose on these points even when the disclosure is negative.

Study description	Data are mixed-methods: quantitative experimental, but also with some qualitative analysis
Research sample	23 healthy adults were recruited as users from the 1SE mobile app, from the local Washington DC area. Participants included 14 female and 9 male adults, mean age=28.3, SD=5.86, range=19 to 37.1 years. We specifically recruited users of the app because of the hundreds of memory videos they have recorded in the app that are central to the study. It would not be possible to obtain such data if recruiting from the general population. However, it is likely this sample was not representative of the general population, because these are individuals with mobile phones who use a specific app and have time to participate in a study. All participants were right-handed and with normal or corrected vision, as part of the inclusion criteria for fMRI studies in our laboratory.
Sampling strategy	Sample size was a convenience sample, we recruited all participants who contacted us from the in-app advertisement on the 1SE mobile app.
Data collection	Behavioral data was collected using MATLAB's Psychtoolbox and a custom web interface. FMRI data was collected at the NIH Clinical Center with a 3 Tesla General Electric MRI scanner system with a 32-channel head coil. There were no between-participant conditions, so condition blinding was not necessary. Only the research team and participant were present at each experiment session.
Timing	Data were collected from June 2017 to December 2018.
Data exclusions	No data were excluded from the analyses.
Non-participation	No participants dropped out of the study after recruitment.
Randomization	There were no experimental groups for this study.

Reporting for specific materials, systems and methods

We require information from authors about some types of materials, experimental systems and methods used in many studies. Here, indicate whether each material, system or method listed is relevant to your study. If you are not sure if a list item applies to your research, read the appropriate section before selecting a response.

Materials & experimental systems

n/a	Involved in the study
☒	☐ Antibodies
☒	☐ Eukaryotic cell lines
☒	☐ Palaeontology and archaeology
☒	☐ Animals and other organisms
☒	☐ Clinical data
☒	☐ Dual use research of concern

Methods

n/a	Involved in the study
☒	☐ ChIP-seq
☒	☐ Flow cytometry
☐	☒ MRI-based neuroimaging

Magnetic resonance imaging

Experimental design

Design type: Event-related design

Design specifications: Participants engaged in 10 runs of 6.3 minutes each, where they saw 30 1-second video clips of their own and 30 1-second video clips of a blind paired participant. Each clip had a 5s ISI and videos were presented in a random order. Participants who had fewer than 300 videos engaged in shorter runs, with 1/10 of their videos shown each run.

Behavioral performance measures: After the scan, participants labeled each video for location, emotion strength, memory strength, and content (familiarity of people and places). They did not make any responses during the scan. The task is generally engaging (watching one's own memories), so we did not exclude any data based on behavioral measures.

Acquisition

Imaging type(s): Functional and structural

Field strength: 3 Tesla

Sequence & imaging parameters: Whole-brain anatomical scans were acquired using the MP2RAGE sequence, with 1mm isotropic voxels. Whole-brain functional scans were acquired with an EPI scan of 2.5mm isotropic voxels (repetition time = 2500ms, echo time = 30ms, flip angle = 75 degrees), with slices aligned parallel to the temporal lobe.

Area of acquisition: A whole-brain scan was used.

Diffusion MRI ☐ Used ☒ Not used

Preprocessing

Preprocessing software: Functional scans were pre-processed with slice timing correction and motion correction using AFNI and surface-based analyses were performed using MATLAB and SUMA. No smoothing was applied.

Normalization: Group contrasts were generated in the surface-space by surface-based alignment to a template surface, and in the volume space by alignment to the MNI template space.

Normalization template: Group contrasts were generated in the surface-space by surface-based alignment to a template surface, and in the volume space by alignment to the MNI template space.

Noise and artifact removal: Motion was regressed out of the general linear model using six regressors (x, y, z, roll, pitch, yaw).

Volume censoring: No volume censoring was conducted.

Statistical modeling & inference

Model type and settings: Our study uses univariate models, multiple regression models, RSA, deep learning, and support vector regression. These are described in detail in the Methods.

Effect(s) tested: We tested the contribution of memory age, strength, distance, emotion, and content to voxel patterns and signal in the brain. We also tested the relationship of these memory properties to each other.

Specify type of analysis: ☐ Whole brain ☐ ROI-based ☒ Both

Anatomical location(s): The hippocampus, parahippocampal cortex, entorhinal cortex, and amygdala were localized anatomically using FreeSurfer's recon-all pipeline.

Statistic type for inference (See Eklund et al. 2016): Voxel-wise

Correction: We use FDR correction, and report some uncorrected measures for exploratory purposes (and this is made clear in the

analysis)

Models & analysis

n/a	Involved in the study
☒	☐ Functional and/or effective connectivity
☒	☐ Graph analysis
☐	☒ Multivariate modeling or predictive analysis

Multivariate modeling and predictive analysis

1) We conducted RSA and multiple regressions to test the ability to predict voxel value (t-statistic) from the memory features mentioned above. Performance was assessed by comparing the beta value (slope) for each predictor versus a null hypothesis of 0 slope.
2) We conducted SVRs to look at ability of visual features in a video to predict memory strength and activity in a memory strength medial parietal cortex region. Trials were randomly split into 80% training and 20% testing, across 25 iterations.
